# Supplementary material for: Recruitment of plasma cells from IL-21-dependent and IL-21-independent immune reactions to the bone marrow
Source: Nat Commun. 2024 May 17;15:4182. doi: 10.1038/s41467-024-48570-0 (PMC11099182; doi:10.1038/s41467-024-48570-0)
Supplement: Supplementary file 1 — Supplementary Information [file 41467_2024_48570_MOESM1_ESM.pdf]

Recruitment of plasma cells from IL21-dependent and IL21-independent immune reactions to the bone marrow

Marta Ferreira-Gomes[#], Yidan Chen[#], Pawel Durek[#], Hector Rincon-Arevalo, Frederik Heinrich, Laura Bauer, Franziska Szelinski, Gabriela Maria Guerra, Ana-Luisa Stefanski, Antonia Niedobitek, Annika Wiedemann, Marina Bondareva, Jacob Ritter, Katrin Lehmann, Sebastian Hardt, Christian Hipfl, Sascha Hein, Eberhard Hildt, Mareen Matz, Henrik E. Mei, Qingyu Cheng, Van Duc Dang, Mario Witkowski, Andreia C. Lino, Andrey Kruglov, Fritz Melchers, Carsten Perka, Eva V. Schrezenmeier, Andreas Hutloff, Andreas Radbruch[$], Thomas Dörner[$], Mir-Farzin Mashreghi[$,]*

#These authors contributed equally
$These authors jointly supervised this work
**corresponding author: mashreghi@drfz.de*

SUPPLEMENTARY INFORMATION

Supplementary Table 1
Demographics and SARS-CoV-2 vaccination status of patients from which bone marrow plasma cells were isolated and analysed. All bone marrow samples were collected during hip replacement surgery. Different analysis were performed based on sample availability, including ELISA of serum.

Bone Marrow Donor ID	Age	Sex	Analysis	SARS-CoV-2 Vaccination status	Days after last vaccine against SARS-CoV-2	Clinical Information	Medication
1681	75	Male	scSeq	unknown	-	Hypertension, Afib, active smoker	pain killer (metamizol, opioid), PPI, DOAC, ASA, ARB, diuretics, cephalosporine antibiotic
1684	50	Male	scSeq	not vaccinated	-	Aortic valve replacement (mechanical), hypertension, hyperlipidemia, adipositas, hypothyroidism	LMW-Heparin, ARB, Ca channel antagonist, beta blocker, levothyroxine, diuretics, pain killer (paracetamol, opioid), statin
538	67	Male	FC	1 time vaccinated	200	Glaucoma	eye drops, skin cream
539	72	Male	FC	2 times vaccinated	153	Hypertension	ACEI, DOAC, beta blocker, statin
541	61	Female	FC, ELISA	2 times vaccinated	44	Hypertension, hypothyroidism	levothyroxin, ARB
542	81	Male	FC	3 times vaccinated	32	CHF (pacemaker)	VKA, beta-blocker, statin, diuretics; pain killer (metamizol)
543	50	Female	FC	2 times vaccinated	138	None	none
544	62	Male	FC	2 times vaccinated	184	None	none
545	67	Female	FC	3 times vaccinated	25	Hypothyroidism, depression, COPD	tricyclic antidepressant, levothyroxin, inhaler (bronchial dilatation), PPI
546	76	Female	ag-specific-scSeq	3 times vaccinated	56	None	none
547	51	Male	ag-specific-scSeq	1 time vaccinated	202	None	none
548	55	Male	ag-specific-scSeq, FC, ELISA	2 times vaccinated	234	Gout	allopurinol
549	83	Female	FC, ELISA	3 times vaccinated	88	Hypertension, hypothyroidism	ACEI, levothyroxine, statin, beta blocker, diuretics
550	55	Male	FC, ELISA	2 times vaccinated	195	Kidney stones, hypertension, prostate hyperplasia	beta blocker, ACEI, tamsulosin
552	52	Male	FC, ELISA	2 times vaccinated	130	None	none
553	43	Female	scSeq, FC	2 times vaccinated	312	Hashimoto thyreoiditis	thyroxin
554	57	Male	FC	3 times vaccinated	35	Hypertension	ACEI, CCB
555	83	Female	scSeq, FC	3 times vaccinated	106	Hypertension, thyroid surgery (hypothyroidism), coronary artery disease	levothyroxin, beta blocker, ACEI, diuretics, ASA, statin

556	78	Female	scSeq, FC, ELISA	3 times vaccinated	71	Hypertension	beta blocker
557	80	Male	FC, ELISA	3 times vaccinated	75	COPD, chronic pain, hypertension	pain killer (metamizol, opioid), inhaler, ARB, gabapentin, Vit D, PPI
558	65	Male	scSeq, FC, ELISA	3 times vaccinated	85	Hypertension, haemochromatosis	ARB, CCB
559	55	Female	scSeq, FC, ELISA	3 times vaccinated	80	Grave's disease, collagen colitis	levothyroxin, tamoxifen, budesonid (topical enteric effect)
561	61	Female	scSeq	1 time vaccinated	188-218	Hypertension, hypothyroidism	levothyroxin, ARB, opioid, PPI
562	37	Female	FC	3 times vaccinated	91	Hypothyroidism, asthma	levothyroxin, inhaler
563	73	Female	FC	3 times vaccinated	132	Hypertension, hypothyroidism	levothyroxin, ACEI, CCB, eye drops

ACEI: angiotensin-converting-enzyme inhibitors; ASA: acetylsalicylic acid; ARB: angiotensin II receptor blocker; CCB: calcium channel blocker; COPD: chronic obstructive pulmonary disease; CHF: chronic heart failure; DOAC: directly acting oral anticoagulant; ELISA: enzyme-linked immunosorbent assay; FC: flow cytometry; PPI: proton-pump inhibitor; scSeq: single cell RNA sequening; VKA: vitamin K antagonist.

Supplementary Table 2

Demographics, vaccination status and analysis time points of patients from which peripheral blood ASC were isolated and analysed. Analysis time points after COVID-19 (different doses) or diphtheria, tetanus, pertussis (DTP) vaccination: **1** - day 7 after 1st dose / **2** - day 14 after 1st dose / **3** - day 7 after 2nd dose / **4** - 7 months after the 2nd dose / **5** - day 7 after 3rd dose / **6** - day 7 after DTP boost / **7** - 6 months after DTP boost.

			COVID-19 Diagnosis	COVID-19 Vaccination					Time between COVID-19 and DTP boost	DTP Vaccination	scSequencing
ID	Age	Sex	Time before analysis	1st dose	Time between doses	2nd dose	Time between doses	3rd dose		Time since previous boost	Analysis time points
11	31	Male	na	Comirnaty	4wks	Comirnaty	9mo	Comirnaty	na	na	1, 2, 4, 5
12	36	Male	na	Comirnaty	3wks	Comirnaty			na	na	1, 2, 3
13	31	Male	na	Comirnaty	3wks	Comirnaty			na	na	1, 2, 3
14	35	Male	na	Comirnaty	4wks	Comirnaty			na	na	1, 2
15	46	Male	na	Comirnaty	3wks	Comirnaty			na	na	1, 2, 3
16	51	Male	na	Comirnaty	3wks	Comirnaty			na	na	1, 2, 3
17	30	Male	na	Comirnaty	4wks	Comirnaty			na	na	1, 2

21	32	Male	na	Comirnaty	3wks	Comirnaty			na	na	1, 2, 3
22	34	Female	na	Comirnaty	3wks	Comirnaty			na	na	1, 2, 3
23	45	Female	na	Comirnaty	3wks	Comirnaty			na	na	1, 2, 3
24	41	Female	na	Comirnaty	3wks	Comirnaty			na	na	1, 2, 3, 4
25	41	Female	na	Comirnaty	3wks	Comirnaty	9mo	Comirnaty	na	na	1, 2, 3, 4, 5
26	27	Male	na	Comirnaty	3wks	Comirnaty			na	na	1, 2, 3
27	39	Female	na	Comirnaty	3wks	Comirnaty	9mo	Comirnaty	na	na	1, 2, 3, 4, 5
28	54	Male	na	Comirnaty	3wks	Comirnaty			na	na	1, 2, 3
31	83	Female	na	Comirnaty	3wks	Comirnaty			na	na	2, 3, 4
32	84	Male	na	Comirnaty	3wks	Comirnaty			na	na	2, 3, 4
33	32	Female	not known	Comirnaty	3wks	Comirnaty			na	na	2, 3
51	42	Female	na	Vaxzevria	12wks	Comirnaty			na	na	1, 2, 3
52	32	Female	na	Vaxzevria	-	-			na	na	1, 2
53	30	Male	na	Vaxzevria	12wks	Comirnaty			na	na	1, 2, 3
54	30	Male	na	Vaxzevria	12wks	Comirnaty			na	na	1, 2, 3
55	31	Male	na	Vaxzevria	12wks	Comirnaty			na	na	1, 2, 3
56	31	Female	na	Vaxzevria	12wks	Comirnaty			na	na	1, 2, 3
57	28	Female	na	Vaxzevria	12wks	Comirnaty			na	na	1, 2, 3
58	36	Male	na	Vaxzevria	12wks	Comirnaty			na	na	1, 2, 3
C1	47	Female	2mo	Comirnaty	-	-			na	na	1
C2	21	Male	4mo	Comirnaty	-	-			na	na	1
C3	34	Female	13mo	Comirnaty	-	-			na	na	1
T1	47	Male	na	Vaxzevria	12wks	Comirnaty			12wks	19yrs	6,7
T2	26	Female	17mo	Vaxzevria	12wks	Comirnaty			19wks	11yrs	6,7
T3	25	Female	12mo	Comirnaty	6wks	Comirnaty			12wks	11yrs	6,7
T4	45	Female	6mo	Vaxzevria	12wks	Comirnaty			21wks	11yrs	7
T5	31	Female	na	Vaxzevria	12wks	Comirnaty			19wks	9yrs	6,7
T6	29	Male	na	Vaxzevria	12wks	Comirnaty			14wks	8yrs	6,7
T7	30	Male	na	Vaxzevria	9wks	Comirnaty			18wks	8yrs	6,7

Supplementary Table 3
Antibody list. List of all used antibodies including clone, conjugate and source information.

Antibody	Clone	Conjugate	Source	Cat No
CD3	BW264/56	VioBlue	Miltenyi Biotec	130-113-133
CD3	UCHT1	FITC	own conjugate	-
CD3	UCHT1	BUV395	BD Biosciences	563546
CD3	HIT3a	PerCP	BioLegend	300326
CD4	91d6	Alexa Fluor 700	own conjugate	-
CD10	97C5	VioBlue	Miltenyi Biotec	130-099-670
CD14	TÜK4	VioBlue	Miltenyi Biotec	130-113-152
CD14	M5E2	BUV395	BD Biosciences	740286
CD19	SJ25C1	BV711	BD Biosciences	563038
CD20	2H7	BV510	BioLegend	302340
CD27	MT271	PE	Miltenyi Biotec	130-113-630
CD27	O323	APC-Cy7	BioLegend	302816
CD27	O323	Brilliant Violet 421	BioLegend	302824
CD27	L128	BV786	BD Biosciences	563328
CD38	HIT2	APC	BioLegend	303510
CD38	HIT2	APC-Cy7	BioLegend	303534
CD38	HIT2	PerCP-Cy5.5	BioLegend	551400
CD38	OKT10	Alexa Fluor 647	own conjugate	-
CD45RA	4G11	FITC	own conjugate	-
CD56	HCD56	BV421	BioLegend	318328
CD138	44F9	PE	Miltenyi Biotec	130-119-840
CD138	MI15	BUV737	BD Biosciences	564393
CXCR5	J252D4	Brilliant Violet 421	BioLegend	356920
HLA-DR	Tü36	PE	BD Biosciences	555561
IgA	G20-359	Biotin	BD Biosciences	555884
IgA	Polyclonal	Biotin	Southern Biotech	2050-08
IgA	Polyclonal	FITC	Southern Biotech	2052-02
IgD	IA6-2	PE/Dazzle594	BioLegend	348240
IgD	IA6-2	APC-Cy7	BioLegend	348218
IgG	G18-145	PE-Cy7	BD Biosciences	561298
IgG	Polyclonal	Alexa647	Southern Biotech	2014-31
IgM	G20-127	BV421	BD Biosciences	562618
PD-1	EH12.2H7	Biotin	BioLegend	329934
SARS-CoV-2 Spike Glycoprotein S1	CR3022		Abcam	ab273073
CD11c	S-HCL-3	TACGCCTATAACTTG	BioLegend	371521
CD19	HIB19	CTGGGCAATTACTCG	BioLegend	302265
CD20	2H7	TTCTGGGTCCCTAGA	BioLegend	302363
CD21	Bu32	AACCTAGTAGTTCGG	BioLegend	354923
CD23	EBVCS-5	TCTGTATAACCGTCT	BioLegend	338525
CD27	O323	GCACTCCTGCATGTA	BioLegend	302853
CD28	CD28.2	TGAGAACGACCCTAA	BioLegend	302963
CD29	TS2/16	GTATTCCCTCAGTCA	BioLegend	303029
CD38	HIT2	TGTACCCGCTTGTGA	BioLegend	303543
CD40	5C3	CTCAGATGGAGTATG	BioLegend	334348
CD44	IM7	TGGCTTCAGGTCCTA	BioLegend	103063
CD45	HI30	TGCAATTACCCGGAT	BioLegend	304068
CD49d	9F10	CCATTCAACTTCCGG	BioLegend	304345
CD49f	GoH3	TTCCGAGGATGATCT	BioLegend	313635
CD56	QA17A16	TTCGCCGCATTGAGT	BioLegend	392425
CD62L	DREG-56	GTCCCTGCAACTTGA	BioLegend	304851
CD66b	6/40c	AGCTGTAAGTTTCGG	BioLegend	392909
CD71	CY1G4	CCGTGTTCCTCATTA	BioLegend	334125
CD73	AD2	CAGTTCCTCAGTTCG	BioLegend	344031
CD79b	CB3-1	ATTCTTCAACCGAAG	BioLegend	341417
CD80	2D10	ACGAATCAATCTGTG	BioLegend	305243
CD86	IT2.2	GTCTTTGTCAGTGCA	BioLegend	305447
CD95	DX2	CCAGCTCATTAGAGC	BioLegend	305651
CD98	MEM-108	GCACCAACAGCCATT	BioLegend	315607
CD107a	H4A3	CAGCCCACTGCAATA	BioLegend	328649
CD138	DL-101	GTATAGACCAAAGCC	BioLegend	352327
CD183	G025H7	GCGATGGTAGATTAT	BioLegend	353747
CD184	12G5	TCAGGTCCTTTCAAC	BioLegend	306533
CD185	J252D4	AATTCAACCGTCGCC	BioLegend	356939
CD268	11C1	CGAAGTCGATCCGTA	BioLegend	316927
CD269	19F2	CAGATGATCCACCAT	BioLegend	357523

CD273	24F.10C12	TCAACGCTTGGCTAG	BioLegend	329621
CD274	29E.2A3	GTTGTCCGACAATAC	BioLegend	329751
CD319	162.1	AGTATGCCATGTCTT	BioLegend	331823
HLA-DR	L243	AATAGCGAGCAAGTA	BioLegend	307663
IgD	IA6-2	CAGTCTCCGTAGAGT	BioLegend	348245
IgM	MHM-88	TAGCGAGCCCGTATA	BioLegend	314547
Integrin β7	FIB504	TCCTTGGATGTACCG	BioLegend	321229
Hashtag 1	LNH-94; 2M2	GTCAACTCTTTAGCG	BioLegend	394661
Hashtag 2	LNH-94; 2M2	TGATGGCCTATTGGG	BioLegend	394663
Hashtag 3	LNH-94; 2M2	TTCCGCCTCTCTTTG	BioLegend	394665
Hashtag 4	LNH-94; 2M2	AGTAAGTTCAGCGTA	BioLegend	394667
Hashtag 5	LNH-94; 2M2	AAGTATCGTTTCGCA	BioLegend	394669
Hashtag 6	LNH-94; 2M2	GGTTGCCAGATGTCA	BioLegend	394671
Hashtag 7	LNH-94; 2M2	TGTCTTTCCTGCCAG	BioLegend	394673
Hashtag 8	LNH-94; 2M2	CTCCTCTGCAATTAC	BioLegend	394675
Hashtag 9	LNH-94; 2M2	CAGTAGTCACGGTCA	BioLegend	394677
Hashtag 10	LNH-94; 2M2	ATTGACCCGCGTTAG	BioLegend	394679
IgG	Polyclonal	HRP	Southern Biotech	2040-05
IgG	Polyclonal	HRP	Cytiva	NA933-1ML

Supplementary Figure 1

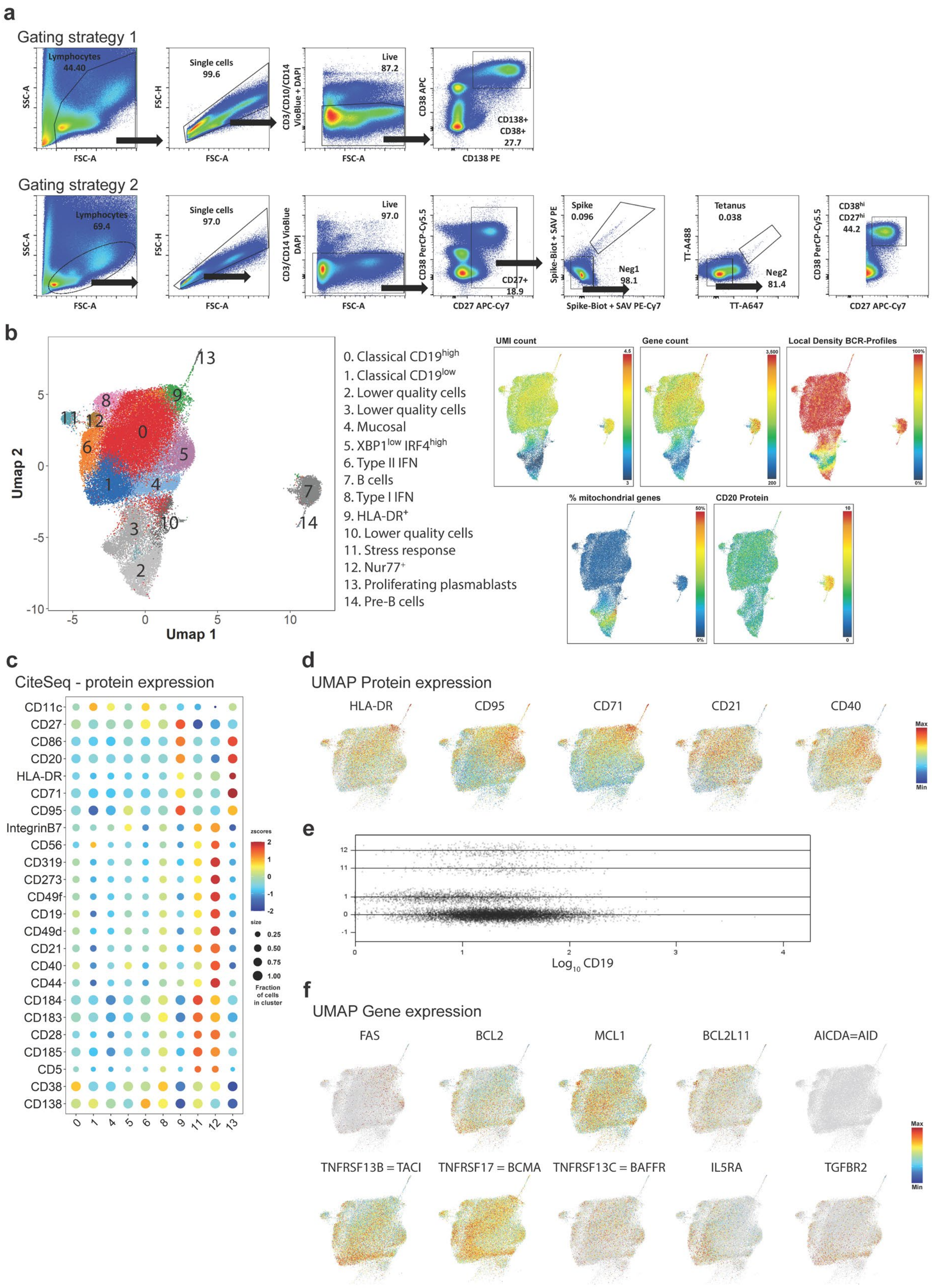

Isolation and analysis of bone marrow plasma cells. (a) Gating strategies used for FACS of BMPC from bone marrow samples (see Supplementary Table 1). From some bone marrow samples, spike-specific and tetanus-specific B cells/plasma cells were additionally sorted (Gating Strategy 2). The same strategy was used to sort antigen-specific cells from peripheral

blood samples. **(b)** UMAP representation of 49347 sorted BMPC from eight bone marrow samples. Clusters of transcriptionally similar cells were identified using shared nearest neighbour (SNN) modularity optimisation. Cluster annotation were manually curated by visual inspection of expression of selected genes, $\log_{10}$ transformed unique molecular identifier (UMI) count (detected mRNA molecules), number of different detected genes, local density of captured BCR sequences, percentage of mitochondrial genes, and surface CD20 expression. Clusters containing a majority of $CD20^{+}$ B cells or lower quality cells were not considered in further analysis. **(c)** Bubble plot of surface protein expression (CITE-Seq) per cluster. Colour scale shows the z-scores of the average expression of a gene within the indicated cluster. Bubble sizes correspond to the fraction of cells expressing a defined gene within the indicated cluster. **(d)** UMAP representation of the expression levels of selected surface proteins (CITE-Seq) by the analysed BMPC. Colour gradient represents the expression levels of the indicated genes. **(e)** Log_{10} transformed CD19 protein surface expression (CITE-Seq) by BMPC of clusters 0, 1, 11 and 12. Representative plot of BMPC from two donors. **(f)** UMAP representation of the expression levels of selected genes by the analysed BMPC. Colour gradient represents the expression levels of the indicated genes. Source data are provided as a Source Data file.

Supplementary Figure 2

a

	0	1	4	5	6	8	9	11	12	13
**0**		3.64	2.28	2.79	1.86	2.25	1.25	0.87	0.74	0.12
**1**	9.91		3.14	2.19	2.06	1.51	0.50	0.35	0.63	0.05
**4**	10.43	5.28		3.25	3.63	1.44	0.93	0.42	0.68	0.00
**5**	15.89	4.58	4.05		2.21	2.16	1.37	1.26	0.68	0.21
**6**	10.93	4.44	4.65	2.27		1.73	1.68	0.70	0.70	0.22
**8**	25.05	6.16	3.49	4.21	3.29		1.85	2.05	1.64	0.00
**9**	16.05	2.38	2.62	3.09	3.69	2.14		0.71	1.07	0.95
**11**	24.16	3.60	2.57	6.17	3.34	5.14	1.54		1.03	0.51
**12**	27.40	8.56	5.48	4.45	4.45	5.48	3.08	1.37		0.34
**13**	11.50	1.77	0.00	3.54	3.54	0.00	7.08	1.77	0.88	

b

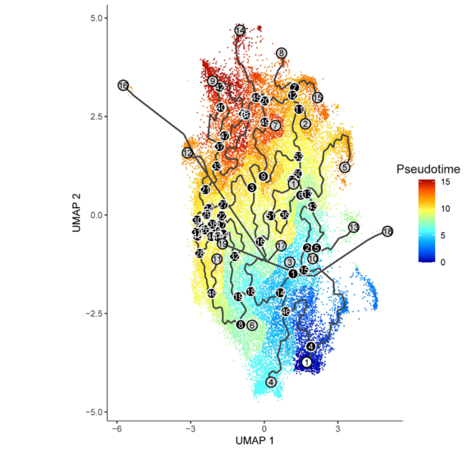

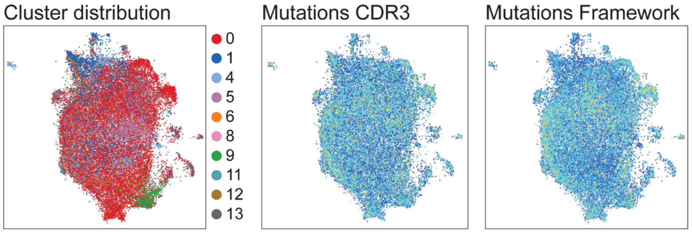

c

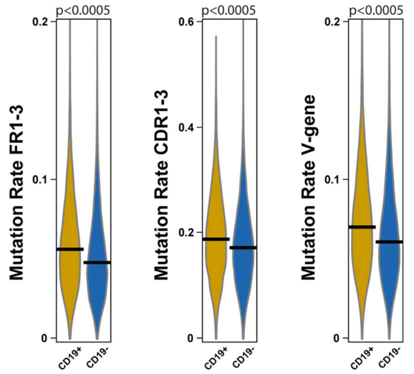

d

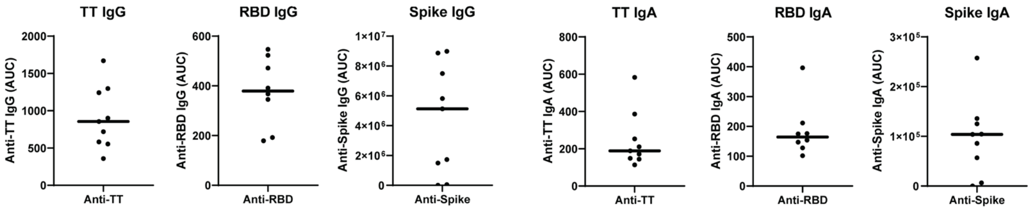

e

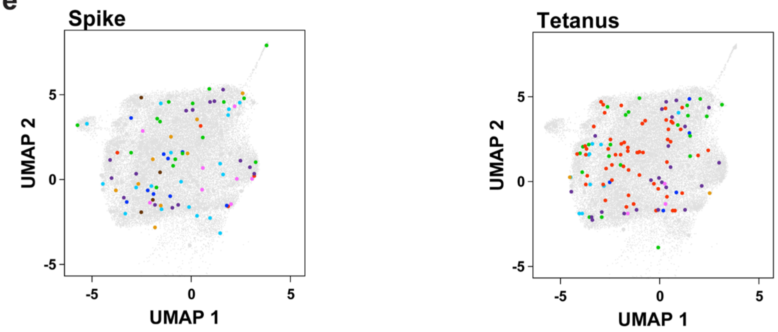

Donor	CD19^high clusters 0	8	9	11	12	13	CD19^low clusters 1	4	5	6	Total
1681	4	1	0	0	0	0	3	1	0	0	9
1684	8	1	1	1	0	0	3	3	0	1	18
553	4	0	1	0	0	0	4	0	0	0	9
555	10	0	1	1	0	1	0	0	1	2	16
556	1	0	0	0	0	0	0	1	1	1	4
558	4	0	1	0	0	0	2	4	2	2	15
559	1	0	1	0	0	0	1	2	2	1	8
561	1	1	0	1	0	0	2	0	0	0	5

Donor	CD19^high clusters 0	8	9	11	12	13	CD19^low clusters 1	4	5	6	Total
1681	1	0	1	0	0	0	1	2	0	0	5
1684	2	0	0	0	0	0	3	0	0	5	10
553	0	0	0	0	0	0	0	1	0	1	2
555	12	0	3	0	0	0	2	1	0	4	22
556	29	5	0	0	0	0	6	8	2	5	55
558	10	0	1	0	0	0	5	2	3	2	23
559	1	0	0	0	0	0	1	1	0	0	3
561	0	0	0	0	0	0	0	0	0	0	0

No relationship or development path among the different BMPC clans. (a) Overlap between BMPC clusters based on clonally related sequences. Numbers correspond row-wise to the percentage of clonal families in the particular cluster co-occurring in a different cluster (column). A clonal family was defined by V and J gene composition and a CDR3 region with

less than 20% Hamming distance in both the heavy and light chains originating from one donor. No statistically significant higher overlaps than expected by 1000 randomisations were found (p-values shown in Supplementary Data 1). Frequencies of clonal families were not considered. **(b)** Single-cell trajectory analysis of 38235 BMPC by Monocle3. Top: UMAP of pseudotime analysis. Cells from cluster 9 (HLA-DR$^+$ newly generated BMPC) were marked as "roots" of the trajectory (white circle). Light grey circles mark areas that correspond to a different outcome (i.e. cell fate) of the trajectory. Black circles indicate branch nodes, in which cells can travel to one of several outcomes. Bottom: Projection of the BMPC clusters depicted in Figure 1a onto the pseudotime analysis UMAP (left). Mutation rates in the CDR3 (middle) and framework regions FR1-3 (right) of the heavy and light chains of the BCRs of BMPC. **(c)** Comparison of mutation rates in the framework regions FR1-3 (left), CDR1-3 regions (middle) or entire V gene (right) of CD19$^+$ and CD19$^-$ BMPC from all clusters. Horizontal lines represent the median mutation rate. Statistics were performed using a two-tailed Mann-Whitney U test. **(d)** Serum titres of TT-, RBD- and spike-specific antibodies from patients from which paired peripheral blood and bone marrow samples could be obtained. Horizontal lines indicate the median. n=9 donors, except for anti-RBD titres where n=8 donors. AUC = area under the curve. **(e)** Identification of SARS-CoV-2 spike-specific and tetanus toxoid-specific public clones among analysed BMPC (see Figure 2e). Each dot in the UMAP represents a public clone and each colour indicates in which particular donor the clone was identified. The tables below show how many public clones were identified per donor per cluster. Source data are provided as a Source Data file.

Supplementary Figure 3

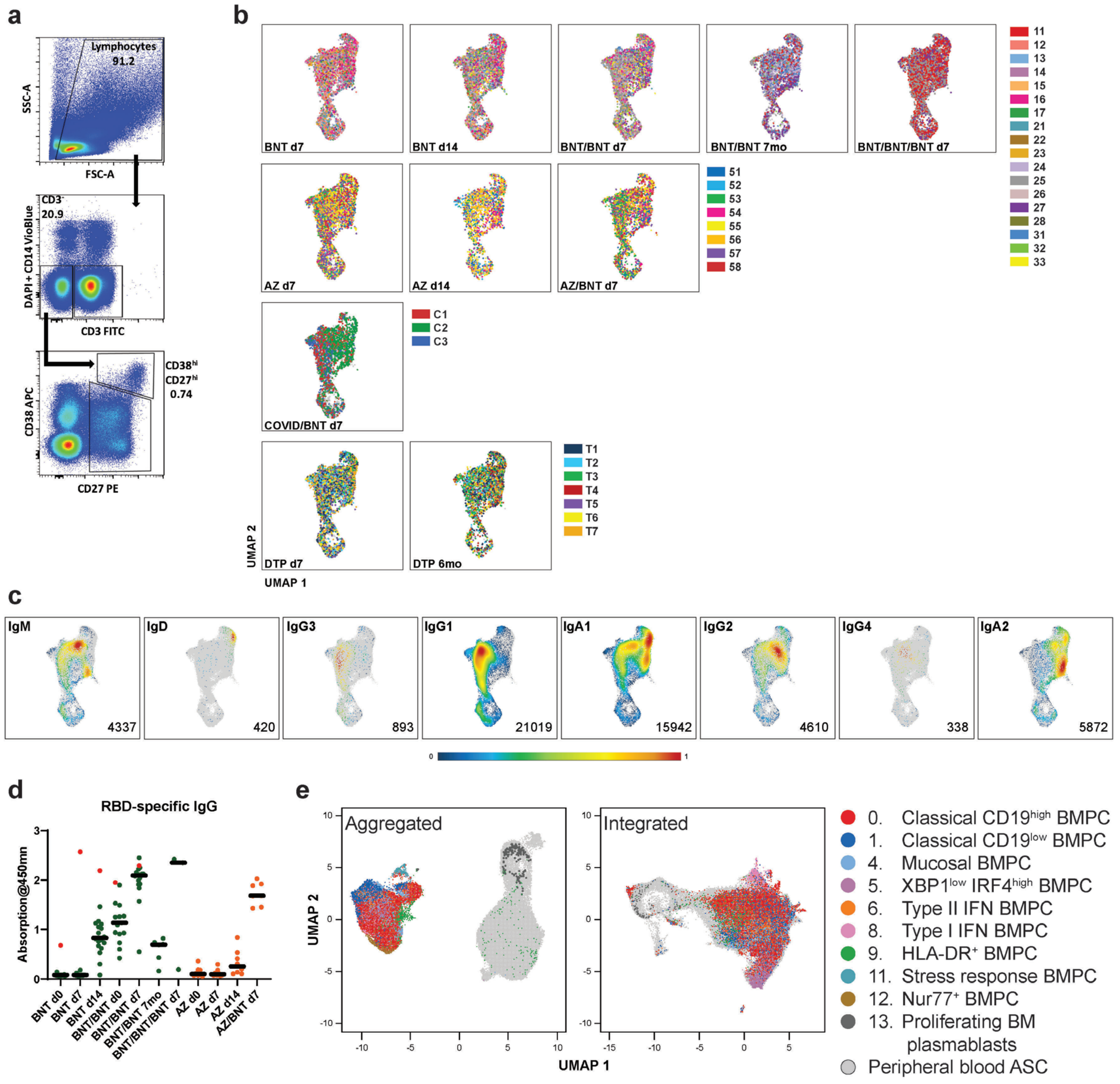

Isolation and analysis of peripheral blood antibody-secreting cells. (a) Gating strategy used for FACS of peripheral blood ASC from individuals who were vaccinated against SARS-CoV-2 or diphtheria, tetanus and pertussis (see Supplementary Table 2). ASC were identified according to $CD38^{high}CD27^{high}CD3^{-}CD14^{-}DAPI^{-}$ expression, after gating on lymphoid cells. **(b)** Distribution of donor's ASC from each time point post vaccination projected onto the obtained UMAP (see Figure 3b). Different colours represent different donors. **(c)** Density plots of immunoglobulin isotype expression of the analysed ASC (determined by BCR sequencing). Shown numbers indicate the number of cells expressing each isotype. **(d)** Serum titres of SARS-COV-2 RBD-specific IgG antibodies from COVID-19 vaccinated individuals through time (1st vaccination dose with BNT or AZ depicted in green or orange, respectively). Depicted in red are the serum titres of a subject which values led to the suspicion of a previous SARS-CoV-2 infection. Horizontal lines indicate the median. BNT d0, d7 and d14, n=18 subjects. BNT/BNT d0, n=15 subjects. BNT/BNT d7, n=18 subjects. BNT/BNT 7mo, n=6 subjects. BNT/BNT/BNT d7, n=3 subjects. AZ d0, d7 and d14, n=8 subjects. AZ/BNT d7, n=7 subjects. **(e)** UMAP representation of the aggregation (left) and additional integration (right) of 38235 BMPC (Figure 1) with 55071 peripheral blood ASC (Figure 3). The identified BMPC clusters

are represented by the respective colours shown in Figure 1. Blood ASC are shown in light grey. Source data are provided as a Source Data file.

Supplementary Figure 4

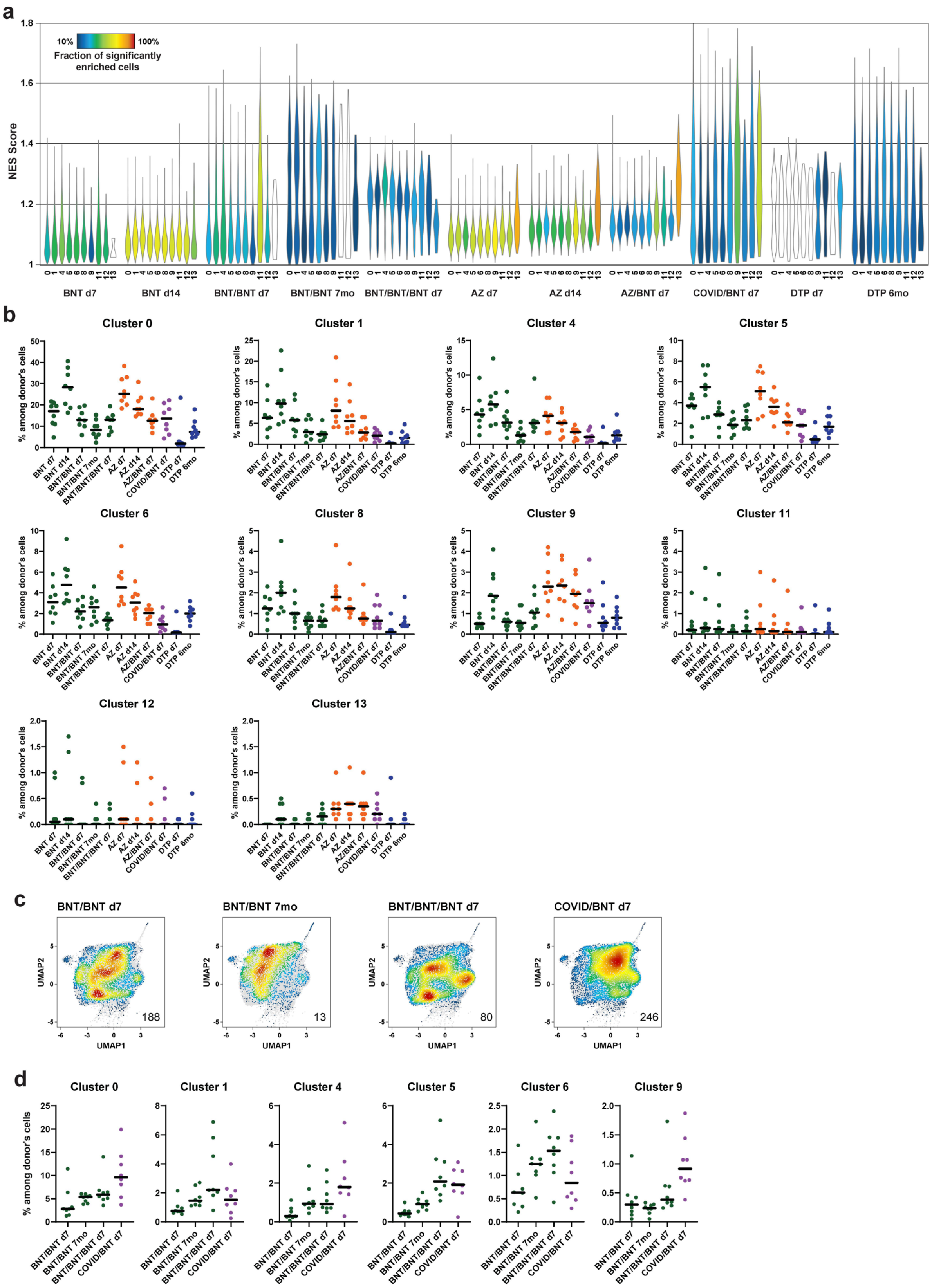

GSEA of peripheral blood ASC gene sets onto BMPC show imprinting of BMPC by the immune response from which they originated. (a) Violin plots of the normalised enrichment score (NES) per BMPC cluster of the GSEA depicted in Figure 3e. Violins are coloured by the fraction of cells per cluster with a significant enrichment. Only clusters with at least 10% of cells were coloured. Statistical significance between NES scores is shown in Supplementary Data 1 (two-tailed Mann-Whitney U test). **(b)** Percentage of positively enriched cells among each donor's total cells per BMPC cluster of the different GSEA depicted in Figure 3e (n=8 donors). **(c)** Density plots of BMPC with significant enrichment identified by GSEA of gene signatures from SARS-CoV-2 spike-specific peripheral blood ASC (public clones) at different time points after BNT vaccination. See Figure 1a for original UMAP projection. Statistical significance between NES scores per cluster is shown in Supplementary Data 1 (two-tailed Mann-Whitney U test). The shown number indicates the number of ASC harbouring public clones used to define gene signatures specific for each time point. **(d)** Percentage of positively enriched cells among each donor's total cells per BMPC cluster of the different GSEA depicted in c (n=8 donors). Only the 5 most predominant clusters are shown. Source data are provided as a Source Data file.

Supplementary Figure 5

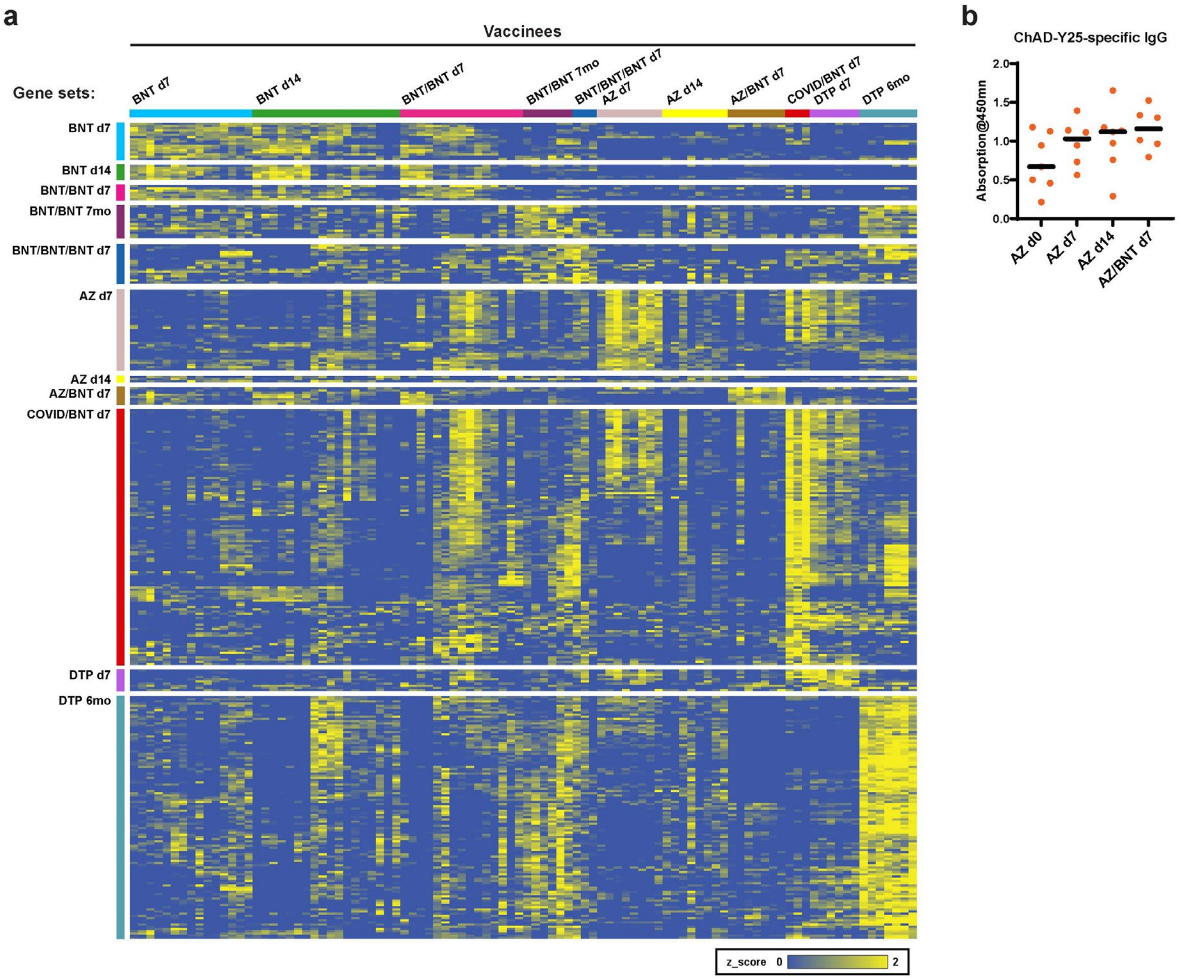

Gene signatures obtained from peripheral blood ASC at different time points after vaccination. (a) Heatmap showing the positive z-score expression of gene sets defining the signature of peripheral blood ASC isolated at different time points after vaccination shown per analysed vaccinee/time post vaccination (see Supplementary Table 2). **(b)** Serum titres of chimpanzee adenovirus Y25 (ChAd Y25)-specific IgG antibodies from Vaxzevria (AZ)

vaccinated individuals over time. Values represent the mean from two replicates. Horizontal lines indicate the median. AZ d0 and d14, n=7 subjects. AZ d7 and AZ/BNT d7, n=6 subjects. Source data are provided as a Source Data file.

Supplementary Figure 6

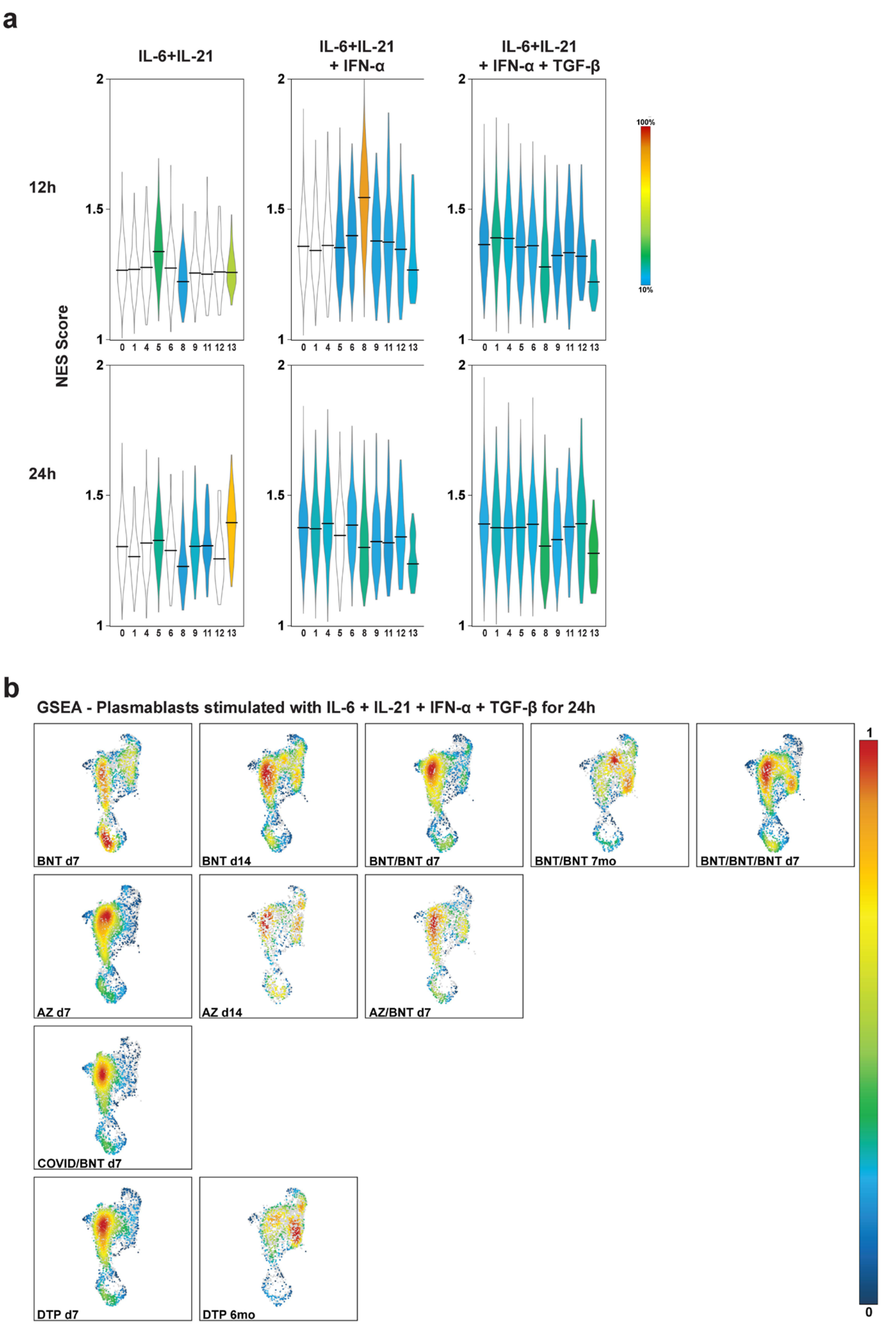

GSEA of stimulated B cell gene sets onto BMPC and ASC show imprinting by the immune response from which they originated. (a) Violin plots of the normalised enrichment score (NES) per BMPC cluster of the GSEA depicted in Figure 4b. Violins are coloured by the fraction of cells per cluster with a significant enrichment. Only clusters with at least 10% of cells were coloured. Statistical significance between NES scores is shown in Supplementary Data 1 (two-tailed Mann-Whitney U test). **(b)** Density plots per time point and vaccine protocol of ASC significantly enriched in gene signatures from *ex vivo*-differentiated plasmablasts

stimulated with IL-6, IL-21, IFN-α and TGF-β for 24 hours as identified by GSEA (data sets from Stephenson *et al.*[1]). See Figure 3b for original UMAP projection. Source data are provided as a Source Data file.

Supplementary Figure 7

a

b

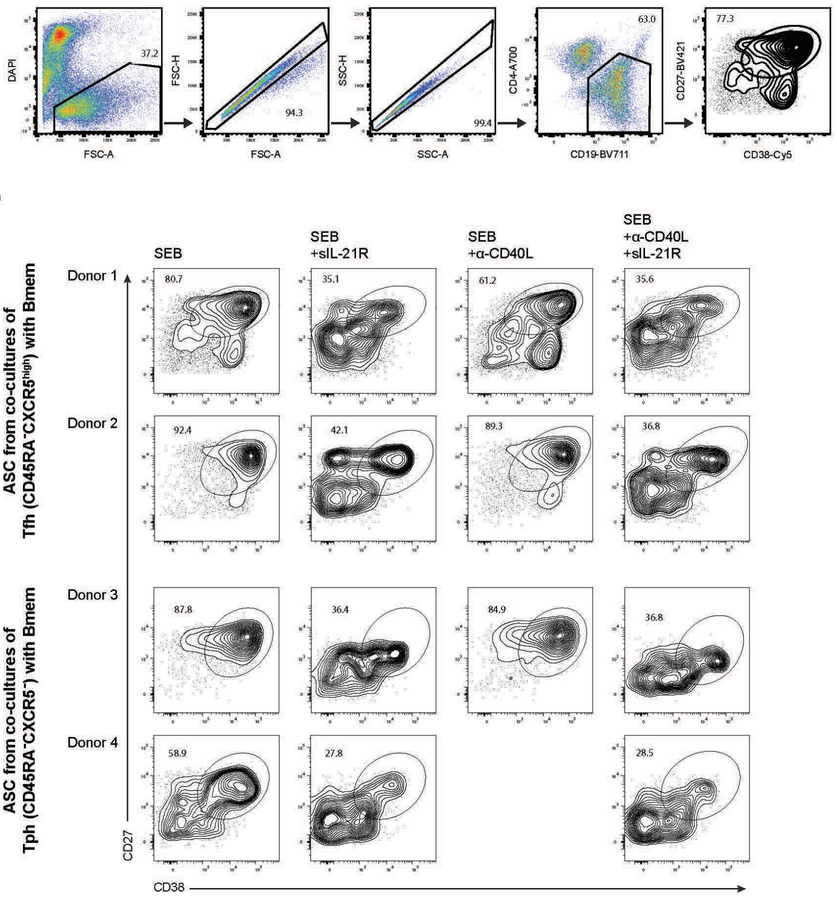

Impact of IL-21 and CD40L in the differentiation of B cells into plasmablasts. (a-b) T follicular helper cells (Tfh, $CD19^-CD4^+CD45RA^-CXCR5^{++}$) from tonsils or T peripheral helper cells (Tph, $CD19^-CD4^+CD45RA^-CXCR5^-$) from BAL of sarcoidosis patients were co-cultured 1:1 with tonsillar memory B cells ($CD19^+CD4^-IgD^-CD38^-$) for 7 days in presence of staphylococcal enterotoxin B (SEB). Where indicated, T cell help was blocked with an anti-CD40L antibody and/or recombinant soluble IL-21R.

(**a**) Gating strategy used for the flow cytometry analysis of differentiated ASC from co-cultures of memory B cells and tonsillar Tfh or BAL Tph in presence of staphylococcal enterotoxin B (SEB) for 7 days. **(b)** Identification of differentiated plasmablasts per donor per co-culture condition.

Supplementary Figure 8

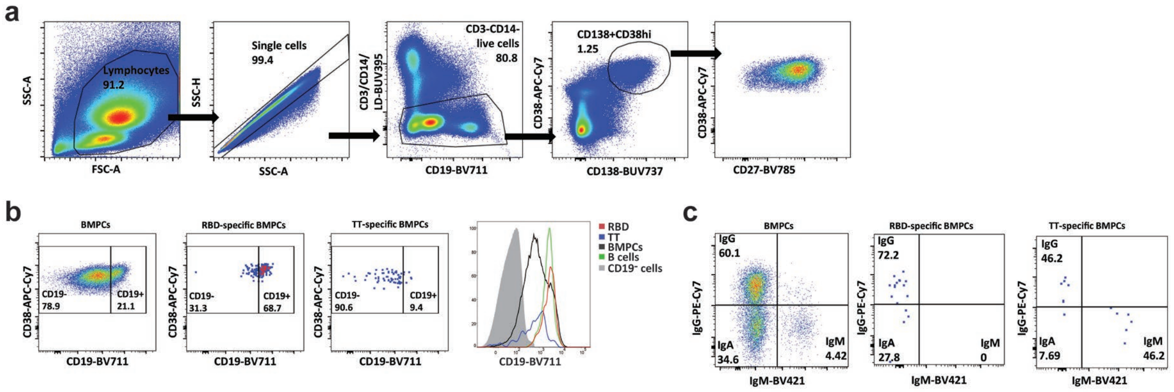

Phenotypic analysis of BMPC. (a) Gating strategy used for the flow cytometry analysis of BMPC (see Supplementary Table 1). BMPC were identified according to $CD138^{+}CD38^{high}CD14^{-}CD3^{-}$ and co-expression of CD27, after gating on lymphoid cells and exclusion of aggregated and dead cells. **(b)** Left: Representative pseudocolour dotplots of CD19 expression in BMPC, RBD-specific BMPC and TT-specific BMPC. Right: Histograms of CD19 staining in RBD-specific (red), TT-specific (blue), total BMPC (black), BM B cells (green) and BM $CD19^{-}$ mononuclear cells (grey). **(c)** Representative pseudocolour dotplots of intracellular IgM and IgG staining in total BMPC, RBD-specific BMPC, and TT-specific BMPC.

References

1 Stephenson, S. *et al.* Growth Factor-like Gene Regulation Is Separable from Survival and Maturation in Antibody-Secreting Cells. *J Immunol* **202**, 1287-1300 (2019). https://doi.org/10.4049/jimmunol.1801407
