# Supplementary material for: Recruitment of plasma cells from IL-21-dependent and IL-21-independent immune reactions to the bone marrow
Source: Nat Commun. 2024 May 17;15:4182. doi: 10.1038/s41467-024-48570-0 (PMC11099182; doi:10.1038/s41467-024-48570-0)
Supplement: Supplementary file 3 — Description of Additional Supplementary Files [file 41467_2024_48570_MOESM3_ESM.pdf]

Description of Additional Supplementary Files

Supplementary Data 1 – description of information in each tab

- Statistics from Fig2d – Statistical significance between V gene mutation rate of the BCRs of BMPC in different clusters. p-values from Mann-Whitney U test.
- Statistics from SupFig2a – Statistics (p-values) from randomization tests for clonal family overlaps between clusters, including Bonferroni correction for multiple testing.
- Statistics from Fig3e/SupFig4a – Statistical significance between NES scores from GSEA of gene signatures from peripheral blood ASC isolated at different time points after vaccination in different BMPC clusters. p-values from Mann-Whitney U test.
- Statistics from SupFig4c – Statistical significance between NES scores from GSEA of gene signatures from SARS-CoV-2 spike-specific peripheral blood ASC (public clones) at different time points after BNT vaccination in different BMPC clusters. p-values from Mann-Whitney U test.
- Statistics from Fig4b/SupFig6a – Statistical significance between NES scores from GSEA of gene signatures from *ex vivo*-differentiated plasmablasts in different BMPC clusters. p-values from Mann-Whitney U test.
- Gene signatures characteristic for the peripheral blood ASC isolated at different time points after Comirnaty (BNT), Vaxzevria (AZ) or Boostrix (DTP) vaccination.
- Gene signatures characteristic for the peripheral blood ASC expressing public clones recognising SARS-CoV-2 RBD/spike protein isolated at different time points after Comirnaty (BNT) vaccination.
- Annotation of the identified putative RBD/spike-specific clones among BMPC, including origin (cellular barcode, donor and cluster), mutation rates, CDR3 sequence and VDJ gene. Heavy chain:Light chain.
- Annotation of the identified putative TT-specific clones among BMPC, including origin (cellular barcode, donor and cluster), mutation rates, CDR3 sequence and VDJ gene. Heavy chain:Light chain.
- Annotation of the identified putative RBD/spike-specific clones among peripheral blood ASC, including origin (cellular barcode, donor and time point of identification), mutation rates, CDR3 sequence and VDJ gene. Heavy chain:Light chain.

- Multiple Sequence Alignment of the identified putative RBD/spike-specific clones among BMPC and peripheral blood ASC with the sequences obtained from sorted RBD/spike-binding memory B cells (highlighted in blue) using CLUSTALW. Putative spike-specific clones were identified by comparison with experimentally validated spike-specific sequences. Each cell (denoted with a cellular barcode ID) exhibits an identity of over 80% to an experimentally validated sequence (highlighted in blue). Sequences are grouped based on consistent lengths of the CDR3. The column "donor" indicates either the donor of a RBD/spike-binding memory B cell sequence or the donor in which a putative RBD/spike-specific clone has been identified. Several individuals in the column "donor" indicate an unclear/ambivalent origin due to pooling of cells from different donors. The column “source” indicates either the ID of the RBD/spike-binding memory B cell sequence or the cell type where the public clone was identified.
- Multiple Sequence Alignment of the identified putative TT-specific clones among BMPC and peripheral blood ASC with the sequences obtained from sorted tetanus toxoid-binding memory B cells (highlighted in blue) using CLUSTALW. Putative TT-specific clones were identified by comparison with experimentally validated TT-specific sequences. Each cell (denoted with a cellular barcode ID) exhibits an identity of over 80% to an experimentally validated sequence (highlighted in blue). Sequences are grouped based on consistent lengths of the CDR3. The column "donor" indicates either the donor of a RBD/spike-binding memory B cell sequence or the donor in which a putative RBD/spike-specific clone has been identified. Several individuals in the column "donor" indicate an unclear/ambivalent origin due to pooling of cells from different donors.
