# Supplementary material for: Recruitment of plasma cells from IL-21-dependent and IL-21-independent immune reactions to the bone marrow
Source: Nat Commun. 2024 May 17;15:4182. doi: 10.1038/s41467-024-48570-0 (PMC11099182; doi:10.1038/s41467-024-48570-0)
Supplement: Supplementary file 5 — Reporting Summary [file 41467_2024_48570_MOESM5_ESM.pdf]

nature portfolio

Corresponding author(s): Mir-Farzin Mashreghi

Last updated by author(s): Apr 16, 2024

Reporting Summary

Nature Portfolio wishes to improve the reproducibility of the work that we publish. This form provides structure for consistency and transparency in reporting. For further information on Nature Portfolio policies, see our Editorial Policies and the Editorial Policy Checklist.

Statistics

For all statistical analyses, confirm that the following items are present in the figure legend, table legend, main text, or Methods section.

n/a	Confirmed	
☐	☒	The exact sample size (*n*) for each experimental group/condition, given as a discrete number and unit of measurement
☐	☒	A statement on whether measurements were taken from distinct samples or whether the same sample was measured repeatedly
☐	☒	The statistical test(s) used AND whether they are one- or two-sided<br>*Only common tests should be described solely by name; describe more complex techniques in the Methods section.*
☒	☐	A description of all covariates tested
☐	☒	A description of any assumptions or corrections, such as tests of normality and adjustment for multiple comparisons
☐	☒	A full description of the statistical parameters including central tendency (e.g. means) or other basic estimates (e.g. regression coefficient) AND variation (e.g. standard deviation) or associated estimates of uncertainty (e.g. confidence intervals)
☐	☒	For null hypothesis testing, the test statistic (e.g. *F*, *t*, *r*) with confidence intervals, effect sizes, degrees of freedom and *P* value noted<br>*Give P values as exact values whenever suitable.*
☒	☐	For Bayesian analysis, information on the choice of priors and Markov chain Monte Carlo settings
☒	☐	For hierarchical and complex designs, identification of the appropriate level for tests and full reporting of outcomes
☐	☒	Estimates of effect sizes (e.g. Cohen's *d*, Pearson's *r*), indicating how they were calculated

Our web collection on statistics for biologists contains articles on many of the points above.

Software and code

Policy information about availability of computer code

Data collection	Flow cytometry data was aquired using the software of the Multi-Application MA900 Cell Sorter (Sony Biotechnology) and FACSDiva (BD Biosciences).
Data analysis	Raw sequence reads were processed using cellranger version 5.0.0. Loupe Browser (version 5, 10x Genomics) was used to identify and define cells of interest by manual gating. Statistics and data analysis was performed in R (version 4.1.2) using the Seurat package (version 4.0.5) and Monocle3.<br>Flow cytometry data was analysed using FlowJo software 10.7.1 (TreeStar).<br>No custom code was used.

For manuscripts utilizing custom algorithms or software that are central to the research but not yet described in published literature, software must be made available to editors and reviewers. We strongly encourage code deposition in a community repository (e.g. GitHub). See the Nature Portfolio guidelines for submitting code & software for further information.

Data

Policy information about availability of data

All manuscripts must include a data availability statement. This statement should provide the following information, where applicable:

- Accession codes, unique identifiers, or web links for publicly available datasets
- A description of any restrictions on data availability
- For clinical datasets or third party data, please ensure that the statement adheres to our policy

Next Generation Sequencing data sets generated in this study are available in the Gene Expression Omnibus (GEO) repository under accession number GSE253862 [https://www.ncbi.nlm.nih.gov/geo/query/acc.cgi?acc= GSE253862]. Data was mapped using the human genome reference hg19 [https://www.10xgenomics.com/support/software/cell-ranger/downloads/cr-ref-build-steps]. The published data sets used for GSEA are available in the Molecular Signatures Database (MSigDB) [https://www.gsea-msigdb.org/gsea/msigdb/]: Hallmark (PMID: 10592173), Reactome (PMID: 29145629) and KEGG (PMID: 10592173); and in the GEO repository under accession number GSE120369 [https://www.ncbi.nlm.nih.gov/geo/query/acc.cgi?acc= GSE120369] (from Stephenson et al. PMID: 30642980). Flow cytometry data files for the analysis of human bone marrow plasma cells are available in the Flow Repository under accession ID FR-FCM-Z7A5 [http://flowrepository.org/id/FR-FCM-Z7A5], while those for the analysis of the co-culture of helper T cells with memory B cells can be found under accession ID FR-FCM-Z7CB [http://flowrepository.org/id/FR-FCM-Z7CB].

Research involving human participants, their data, or biological material

Policy information about studies with human participants or human data. See also policy information about sex, gender (identity/presentation), and sexual orientation and race, ethnicity and racism.

Reporting on sex and gender

Participant's sex as assigned. Due to the small number of analysed samples, no disaggregated sex or gender analysis was performed.

Reporting on race, ethnicity, or other socially relevant groupings

We have not collected information on on race, ethnicity, or other socially relevant groupings.

Population characteristics

Bone Marrow sample donors:
Donor ID 1681, age 75, Male, unknown SARS-CoV-2 vaccination status;
Donor ID 1684, age 50, Male, not vaccinated against SARS-CoV-2;
Donor ID 538, age 67, Male, 1 time vaccinated against SARS-CoV-2, analysis 200 days after vaccination;
Donor ID 539, age 72, Male, 2 times vaccinated against SARS-CoV-2, analysis 153 days after vaccination;
Donor ID 541, age 61, Female, 2 times vaccinated against SARS-CoV-2, analysis 44 days after vaccination;
Donor ID 542, age 81, Male, 3 times vaccinated against SARS-CoV-2, analysis 32 days after vaccination;
Donor ID 543, age 50, Female, 2 times vaccinated against SARS-CoV-2, analysis 138 days after vaccination;
Donor ID 544, age 62, Male, 2 times vaccinated against SARS-CoV-2, analysis 184 days after vaccination;
Donor ID 545, age 67, Female, 3 times vaccinated against SARS-CoV-2, analysis 25 days after vaccination;
Donor ID 546, age 76, Female, 3 times vaccinated against SARS-CoV-2, analysis 56 days after vaccination;
Donor ID 547, age 51, Male, 1 time vaccinated against SARS-CoV-2, analysis 202 days after vaccination;
Donor ID 548, age 55, Male, 2 times vaccinated against SARS-CoV-2, analysis 234 days after vaccination;
Donor ID 549, age 83, Female, 3 times vaccinated against SARS-CoV-2, analysis 88 days after vaccination;
Donor ID 550, age 55, Male, 2 times vaccinated against SARS-CoV-2, analysis 195 days after vaccination;
Donor ID 552, age 52, Male, 2 times vaccinated against SARS-CoV-2, analysis 130 days after vaccination;
Donor ID 553, age 43, Female, 2 times vaccinated against SARS-CoV-2, analysis 312 days after vaccination;
Donor ID 554, age 57, Male, 3 times vaccinated against SARS-CoV-2, analysis 35 days after vaccination;
Donor ID 555, age 83, Female, 3 times vaccinated against SARS-CoV-2, analysis 106 days after vaccination;
Donor ID 556, age 78, Female, 3 times vaccinated against SARS-CoV-2, analysis 71 days after vaccination;
Donor ID 557, age 80, Male, 3 times vaccinated against SARS-CoV-2, analysis 75 days after vaccination;
Donor ID 558, age 65, Male, 3 times vaccinated against SARS-CoV-2, analysis 85 days after vaccination;
Donor ID 559, age 55, Female, 3 times vaccinated against SARS-CoV-2, analysis 80 days after vaccination;
Donor ID 561, age 61, Female, 1 time vaccinated against SARS-CoV-2, analysis 188-218 days after vaccination;
Donor ID 562, age 37, Female, 3 times vaccinated against SARS-CoV-2, analysis 91 days after vaccination;
Donor ID 563, age 73, Female, 3 times vaccinated against SARS-CoV-2, analysis 132 days after vaccination.

Vaccination subjects:
Comirnaty homologous vaccination:
Subject ID 11, age 31, Male, 4wks between doses, 3rd Comirnaty dose after 9 months;
Subject ID 12, age 36, Male, 3wks between doses;
Subject ID 13, age 31, Male, 3wks between doses;
Subject ID 14, age 35, Male, 4wks between doses;
Subject ID 15, age 46, Male, 3wks between doses;
Subject ID 16, age 51, Male, 3wks between doses;
Subject ID 17, age 30, Male, 4wks between doses;
Subject ID 21, age 32, Male, 3wks between doses;
Subject ID 22, age 34, Female, 3wks between doses;
Subject ID 23, age 45, Female, 3wks between doses;

Subject ID 24, age 41, Female, 3wks between doses;
Subject ID 25, age 41, Female, 3wks between doses, 3rd Comirnaty dose after 9 months;
Subject ID 26, age 27, Male, 3wks between doses;
Subject ID 27, age 39, Female, 3wks between doses, 3rd Comirnaty dose after 9 months;
Subject ID 28, age 54, Male, 3wks between doses;
Subject ID 31, age 83, Female, 3wks between doses;
Subject ID 32, age 84, Male, 3wks between doses;
Subject ID 33, age 32, Female, 3wks between doses.

Vaxzevria/Comirnaty heterologous vaccination:
Subject ID 51, age 42, Female, 12wks between doses;
Subject ID 52, age 32, Female, subject dropped out after 1st dose;
Subject ID 53, age 30, Male, 12wks between doses;
Subject ID 54, age 30, Male, 12wks between doses;
Subject ID 55, age 31, Male, 12wks between doses;
Subject ID 56, age 31, Female, 12wks between doses;
Subject ID 57, age 28, Female, 12wks between doses;
Subject ID 58, age 36, Male, 12wks between doses.

Comirnaty vaccination after COVID-19 diagnosis
Subject ID C1, age 47, Female, vaccination 2mo after COVID-19 diagnosis;
Subject ID C2, age 21, Male, vaccination 4mo after COVID-19 diagnosis;
Subject ID C3, age 34, Female, vaccination 13mo after COVID-19 diagnosis.

Boostrix vaccination
Subject ID T1, age 47, Male, previous tetanus vaccination 19yrs before, COVID-19 vaccination 12wks before;
Subject ID T2, age 26, Female, previous tetanus vaccination 11yrs before, COVID-19 vaccination 19wks before;
Subject ID T3, age 25, Female, previous tetanus vaccination 11yrs before, COVID-19 vaccination 12wks before;
Subject ID T5, age 31, Female, previous tetanus vaccination 9yrs before, COVID-19 vaccination 19wks before;
Subject ID T6, age 29, Male, previous tetanus vaccination 8yrs before, COVID-19 vaccination 14wks before;
Subject ID T7, age 30, Male, previous tetanus vaccination 8yrs before, COVID-19 vaccination 18wks before.

Recruitment	Recruitment of bone marrow sample donors was determined by the scheduling of hip arthroplasty surgeries at the time of study. Samples were processed each time a sample from a consenting patient was allocated to our working group by the Charité Orthopedics Surgery Department. Samples from patients with underlying malignant or inflammatory disease were not processed. Due to the nature of the surgery, there is a bias towards an older population (median age of 62), often with age-associated co-morbidities such as hypertension. However, since we see no differences in bone marrow plasma cell populations across all analysed subjects independently of their age, this does not show any impact in the results of this study. Recruitment of vaccination study participants was determined by the number of volunteers who were able to be vaccinated at the time of the study (early COVID-19 vaccination campaign in Berlin, Germany) and agreed to donate blood at defined time points after vaccination. Recruitment of tonsils sample donors was determined by the scheduling of surgeries at the time of study. Recruitment of BAL sample donors was determined by the sample collection at the time of study. There were no self-selection bias.
Ethics oversight	Ethics Committee of the Charité Universitätsmedizin Berlin in compliance with the Declaration of Helsinki (EA1/261/09). Informed consent was obtained from all donors included in the study.

Note that full information on the approval of the study protocol must also be provided in the manuscript.

Field-specific reporting

Please select the one below that is the best fit for your research. If you are not sure, read the appropriate sections before making your selection.

☒ Life sciences ☐ Behavioural & social sciences ☐ Ecological, evolutionary & environmental sciences

For a reference copy of the document with all sections, see nature.com/documents/nr-reporting-summary-flat.pdf

Life sciences study design

All studies must disclose on these points even when the disclosure is negative.

Sample size	Bone marrow, tonsils and BAL sample size was determined by the availability of patient material at the time of study. Number of vaccination study participants was determined by the number of volunteers.
Data exclusions	Single-cell sequencing data from one Boostrix vaccinee (T4) 7 days after boost was excluded due to a COVID-19 diagnosis one day after sample collection.
Replication	Samples processed for single cell sequencing were processed independently or in small batches, with the results from the analysis of the different subjects being consistent. The in vitro differentiation of plasmablasts was replicated four times with different subjects.

Randomization

Due to the analysis of each sample type in a single group, no randomization was applicable. The bone marrow plasma study cohort consisted only of patients undergoing total hip arthroplasty. In the vaccination study cohorts, all participants received the vaccine; there were no unvaccinated controls.

Blinding

Blinding was not applicable due to the nature of the samples analyzed. The bone marrow plasma study cohort consisted only of patients undergoing total hip arthroplasty and in the vaccination study cohorts, all participants received the vaccine; there were no unvaccinated controls. The scientists performing bioinformatics analysis or antibody titre determination did not have patient information into consideration. Clinical history was only obtained after analysis.

Reporting for specific materials, systems and methods

We require information from authors about some types of materials, experimental systems and methods used in many studies. Here, indicate whether each material, system or method listed is relevant to your study. If you are not sure if a list item applies to your research, read the appropriate section before selecting a response.

Materials & experimental systems

n/a	Involved in the study
☐	☒ Antibodies
☐	☒ Eukaryotic cell lines
☒	☐ Palaeontology and archaeology
☒	☐ Animals and other organisms
☒	☐ Clinical data
☒	☐ Dual use research of concern
☒	☐ Plants

Methods

n/a	Involved in the study
☒	☐ ChIP-seq
☐	☒ Flow cytometry
☒	☐ MRI-based neuroimaging

Antibodies

Antibodies used

Flow cytometry anti-human antibodies:
CD3, clone BW264/56, VioBlue, Miltenyi Biotec, Cat. 130-113-133; CD3, clone UCHT1, FITC, DRFZ in-house; CD3, clone UCHT1, BUV395, BD Biosciences, Cat. 563546; CD3, clone HIT3a, PerCP, BioLegend, Cat. 300326; CD4, clone 91d6, Alexa Fluor 700, own conjugate; CD10, clone 97C5, VioBlue, Miltenyi Biotec, Cat. 130-099-670; CD14, clone TÜK4, VioBlue, Miltenyi Biotec, Cat. 130-113-152; CD14, clone M5E2, BUV395, BD Biosciences, Cat. 740286; CD19, clone SJ25C1, BV711, BD Biosciences, Cat. 563038; CD20, clone 2H7, BV510, BioLegend, Cat. 302340; CD27, clone MT271, PE, Miltenyi Biotec, Cat. 130-113-630; CD27, clone O323, APC-Cy7, BioLegend, Cat. 302816; CD27, clone O323, Brilliant Violet 421, BioLegend, Cat. 302824; CD27, clone L128, BV786, BD Biosciences, Cat. 563328;
CD38, clone HIT2, APC, BioLegend, Cat. 303510; CD38, clone HIT2, APC-Cy7, BioLegend, Cat. 303534; CD38, clone HIT2, PerCP-Cy5.5, BioLegend, Cat. 551400; CD38, clone OKT10, Alexa Fluor 647, own conjugate; CD45RA, clone 4G11, FITC, own conjugate; CD56, clone HCD56, BV421, BioLegend, Cat. 318328; CD138, clone 44F9, PE, Miltenyi Biotec, Cat. 130-119-840; CD138, clone MI15, BUV737, BD Biosciences, Cat. 564393; CXCR5 , clone J252D4, Brilliant Violet 421, BioLegend, Cat. 356920; HLA-DR, clone Tü36, PE, BD Biosciences, Cat. 555561;
IgA, clone G20-359, Biotin, BD Biosciences, Cat. 555884; IgA, Polyclonal, FITC, Southern Biotech, Cat. 2052-02; IgD, clone IA6-2, PE/Dazzle594, BioLegend, Cat. 348240; IgD, clone IA6-2, APC-Cy7, BioLegend, Cat. 348218; IgG, clone G18-145, PE-Cy7, BD Biosciences, Cat. 561298; IgG, Polyclonal, Alexa647, Southern Biotech, Cat. 2014-31; IgM, clone G20-127, BV421, BD Biosciences, Cat. 562618;
PD-1, clone EH12.2H7, Biotin/Streptavidin-PE-Cy7 BioLegend 329934.

CITE-Seq anti-human antibodies:
CD11c, clone S-HCL-3, TACGCCTATAACTTG, BioLegend, Cat. 371521; CD19, clone HIB19, CTGGGCAATTACTCG, BioLegend, Cat. 302265; CD20, clone 2H7, TTCTGGGTCCCTAGA, BioLegend, Cat. 302363; CD21, clone Bu32, AACCTAGTAGTTCGG, BioLegend, Cat. 354923; CD23, clone EBVCS-5, TCTGTATAACCGTCT, BioLegend, Cat. 338525; CD27, clone O323, GCACTCCTGCATGTA, BioLegend, Cat. 302853; CD28, clone CD28.2, TGAGAACGACCCTAA, BioLegend, Cat. 302963; CD29, clone TS2/16, GTATTCCCTCAGTCA, BioLegend, Cat. 303029; CD38, clone HIT2, TGTACCCGCTTGTGA, BioLegend, Cat. 303543; CD40, clone 5C3, CTCAGATGGAGTATG, BioLegend, Cat. 334348; CD44, clone IM7, TGGCTTCAGGTCCTA, BioLegend, Cat. 103063; CD45, clone HI30, TGCAATTACCCGGAT, BioLegend, Cat. 304068; CD49d, clone 9F10, CCATTCAACTTCCGG, BioLegend, Cat. 304345; CD49f, clone GoH3, TTCCGAGGATGATCT, BioLegend, Cat. 313635; CD56, clone QA17A16, TTCGCCGCATTGAGT, BioLegend, Cat. 392425; CD62L, clone DREG-56, GTCCCTGCAACTTGA, BioLegend, Cat. 304851; CD66b, clone 6/40c, AGCTGTAAGTTTCGG, BioLegend, Cat. 392909; CD71, clone CY1G4, CCGTGTTCCTCATTA, BioLegend, Cat. 334125; CD73, clone AD2, CAGTTCCTCAGTTCG, BioLegend, Cat. 344031; CD79b, clone CB3-1, ATTCTTCAACCGAAG, BioLegend, Cat. 341417; CD80, clone 2D10, ACGAATCAATCTGTG, BioLegend, Cat. 305243; CD86, clone IT2.2, GTCTTTGTCAGTGCA, BioLegend, Cat. 305447; CD95, clone DX2, CCAGCTCATTAGAGC, BioLegend, Cat. 305651; CD98, clone MEM-108, GCACCAACAGCCATT, BioLegend, Cat. 315607; CD107a, clone H4A3, CAGCCCACTGCAATA ,BioLegend, Cat. 328649; CD138, clone DL-101, GTATAGACCAAAGCC, BioLegend, Cat. 352327; CD183, clone G025H7, GCGATGGTAGATTAT, BioLegend, Cat. 353747; CD184, clone 12G5, TCAGGTCCTTTCAAC, BioLegend, Cat. 306533; CD185, clone J252D4, AATTCAACCGTCGCC, BioLegend, Cat. 356939; CD268, clone 11C1, CGAAGTCGATCCGTA, BioLegend, Cat. 316927; CD269, clone 19F2, CAGATGATCCACCAT, BioLegend, Cat. 357523; CD273, clone 24F.10C12, TCAACGCTTGGCTAG, BioLegend, Cat. 329621; CD274, clone 29E.2A3, GTTGTCCGACAATAC, BioLegend, Cat. 329751; CD319, clone 162.1, AGTATGCCATGTCTT, BioLegend, Cat. 331823; HLA-DR, clone L243, AATAGCGAGCAAGTA, BioLegend, Cat. 307663; IgD, clone IA6-2, CAGTCTCCGTAGAGT, BioLegend, Cat. 348245; IgM, clone MHM-88, TAGCGAGCCCGTATA, BioLegend, Cat. 314547; Integrin β7, clone FIB504, TCCTTGGATGTACCG, BioLegend, Cat. 321229; Hashtag 1, clone LNH-94; 2M2, GTCAACTCTTTAGCG, BioLegend, Cat. 394661; Hashtag 2, clone LNH-94; 2M2, TGATGGCCTATTGGG, BioLegend, Cat. 394663; Hashtag

3, clone LNH-94; 2M2, TTCCGCCTCTCTTTG, BioLegend, Cat. 394665; Hashtag 4, clone LNH-94; 2M2, AGTAAGTTCAGCGTA, BioLegend, Cat. 394667; Hashtag 5, clone LNH-94; 2M2, AAGTATCGTTTCGCA, BioLegend, Cat. 394669; Hashtag 6, clone LNH-94; 2M2, GGTTGCCAGATGTCA, BioLegend, Cat. 394671; Hashtag 7, clone LNH-94; 2M2, TGTCTTTCCTGCCAG, BioLegend, Cat. 394673; Hashtag 8, clone LNH-94; 2M2, CTCCTCTGCAATTAC, BioLegend, Cat. 394675; Hashtag 9, clone LNH-94; 2M2, CAGTAGTCACGGTCA, BioLegend, Cat. 394677; Hashtag 10, clone LNH-94; 2M2, ATTGACCCGCGTTAG, BioLegend , Cat. 394679.

ELISA detection anti-human antibodies:
IgA, Polyclonal, Biotin, Southern Biotech, Cat. 2050-08; IgG, Polyclonal, HRP, Southern Biotech, Cat. 2040-05; IgG, Polyclonal, HRP, Cytiva, Cat. NA933-1ML.

Validation

All purchased antibodies were validated by their manufacturers and further in-house testing.

Miltenyi Biotec
"All our antibodies are rigorously tested and validated before release. In the application section on the product page, you can find examples of typical performance data. In addition, we provide extended validation data highlighting details of antibody performance, specificity, and fixation compatibility. All antibodies for which any of these datasets are already available will be indicated with the extended validation stamp."
Validation of antibody specificity by:
- Counterstaining
- Knockout of target protein
- Epitope competition assay
- siRNA knockdown
- Stimulation of cells
- Overexpression of target protein
- Binding to purified antigen (latex bead coating)
- Cross-reactivity

BioLegend Flow Cytometry Reagents
"Specificity testing of 1-3 target cell types with either single- or multi-color analysis (including positive and negative cell types).
Once specificity is confirmed, each new lot must perform with similar intensity to the in-date reference lot. Brightness (MFI) is evaluated from both positive and negative populations.
Each lot product is validated by QC testing with a series of titration dilutions."

BioLegend TotalSeq™ Antibodies
"Bulk lots are tested by PCR and sequencing to confirm the oligonucleotide barcodes. They are also tested by flow cytometry to ensure the antibodies recognize the proper cell populations.
Bottled lots are tested by PCR and sequencing to confirm the oligonucleotide barcodes."

BD Biosciences
"The specificity is confirmed by using multiple applications that may include a combination of flow cytometry, immunofluorescence, immunohistochemistry or western blot to test a combination of primary cells, cell lines or transfectant models."
"Once our research and development (R&D) team completes evaluation of a new product, the developed process is transferred to our manufacturing teams, including Quality Control."
"Quality control testing of new, manufactured lots are performed side-by-side with a previously accepted lot as a control, helping to serve as a reference for comparison and assuring that performance of the new lot is both reliable and consistent."
"Our strict adherence to these guidelines helps ensure that different lots of conjugated reagents are performing consistently."

Southern Biotech
"SouthernBiotech anti-human secondary antibodies are extensively characterized, affinity-purified polyclonal or monoclonal antibodies with specificities to the human immunoglobulin heavy chain classes and subclasses, light chains, and fragments."
"Our polyclonal anti-human secondary antibodies can provide improved sensitivity through signal amplification as multiple secondary antibodies can bind to a single primary antibody. Selected antibodies have been cross-adsorbed against IgG and/or serum proteins from other species to generate antibodies with minimal cross-reactivity to those species."

Cytiva
"The antibodies are prepared by hyper-immunizing an animal with purified immunoglobulin fractions from normal human serum to produce high affinity antibodies. The pooled antiserum is used to produce an immunoglobulin preparation that is then affinity adsorbed to remove cross-reacting antibodies with the immunoglobulins of other species. These activities are thoroughly depleted to ensure species-specificity. Finally, to select for specific binding to human IgG, the antibodies are purified using an affinity column of human IgG. After washing to remove non-specific serum components and low affinity antibodies, the species-specific antibodies are eluted using carefully selected, mild conditions that minimize aggregation and preserve immunological activity, yet which will elute high affinity antibodies."

Eukaryotic cell lines

Policy information about cell lines and Sex and Gender in Research

Cell line source(s)	HEK293T cells from ATCC (293T (ATCC® CRL-3216™))
Authentication	From vendor. ATCC authenticates cell lines routinely by STR profiling, cellular morphology, karyotyping and cytochrome C oxidase I assay.

Mycoplasma contamination	Cells were not tested for mycoplasma contamination during the time of the study.
Commonly misidentified lines (See ICLAC register)	No commonly misidentified lines were used

Plants

Seed stocks	*Report on the source of all seed stocks or other plant material used. If applicable, state the seed stock centre and catalogue number. If plant specimens were collected from the field, describe the collection location, date and sampling procedures.*
Novel plant genotypes	*Describe the methods by which all novel plant genotypes were produced. This includes those generated by transgenic approaches, gene editing, chemical/radiation-based mutagenesis and hybridization. For transgenic lines, describe the transformation method, the number of independent lines analyzed and the generation upon which experiments were performed. For gene-edited lines, describe the editor used, the endogenous sequence targeted for editing, the targeting guide RNA sequence (if applicable) and how the editor was applied.*
Authentication	*Describe any authentication procedures for each seed stock used or novel genotype generated. Describe any experiments used to assess the effect of a mutation and, where applicable, how potential secondary effects (e.g. second site T-DNA insertions, mosiacism, off-target gene editing) were examined.*

Flow Cytometry

Plots

Confirm that:

- [x] The axis labels state the marker and fluorochrome used (e.g. CD4-FITC).
- [x] The axis scales are clearly visible. Include numbers along axes only for bottom left plot of group (a 'group' is an analysis of identical markers).
- [x] All plots are contour plots with outliers or pseudocolor plots.
- [x] A numerical value for number of cells or percentage (with statistics) is provided.

Methodology

Sample preparation

Bone marrow samples for cell sorting:
Samples were fragmented and transferred to a 50mL tube where they were vortexed to separate cells from bone fragments. Samples were subsequently rinsed through a 70µM filter with PBS/1% BSA/5mM EDTA/2µg/mL actinomycin D to obtain a cell suspension. Plasma cells were enriched from bone marrow using StraightFrom Whole Blood and Bone Marrow CD138 MicroBeads and StraightFrom Whole Blood CD19 MicroBeads (Miltenyi Biotec) according to manufacturer's instructions. Enriched cells were incubated with Fc Blocking Reagent (Miltenyi Biotec) following manufacturer's instructions and subsequently stained for 30 min at 4°C with fluorophore-coupled anti-human antibodies and/or fluorophore-coupled proteins. To stop the staining, cells were washed with PBS/1%BSA. DAPI was added before sorting to allow dead cell exclusion.

Bone marrow samples for flow cytometry analysis:
Samples were fragmented and transferred to a 50mL tube where they were vortexed to separate cells from bone fragments. Samples were subsequently rinsed through a 70µM filter with PPBS/0.5%BSA/EDTA (PBE) to obtain a cell suspension. Mononuclear cells were enriched by density gradient centrifugation over Ficoll-Paque PLUS (GE Healthcare Bio-Sciences). The collected mononuclear cells were filtered with a 70 µm cell strainer (BD Biosciences) and then washed twice with PBE. For staining, LIVE/DEAD Fixable Blue Dead Cell Stain Kit (ThermoFisher Scientific) was used to exclude dead cells according to the manufacturer's protocol. Cells were surface-stained for 30 min at 4°C with fluorophore-coupled anti-human antibodies diluted in Brilliant Stain buffer (BD Horizon). For intracellular staining, cells were washed twice with PBE, fixed for 20 min at 4°C using Fixation/Permeabilization Solution Kit (BD Cytofix/Cytoperm™ Plus) and washed twice with perm/wash buffer. Cells were then incubated for 30 min 4°C with fluorophore-coupled anti-human antibodies and fluorophore-coupled proteins. To stop the staining, cells were washed with PBE.

Peripheral blood samples for cell sorting:
Lymphocytes were enriched from peripheral blood using StraightFrom Whole Blood CD19 and CD3 MicroBeads and StraightFrom Whole Blood and Bone Marrow CD138 MicroBeads (Miltenyi Biotec) according to manufacturer's instructions. 2µg/mL actinomycin D was added to the buffer used during the first centrifugation. Enriched cells were incubated with Fc Blocking Reagent (Miltenyi Biotec) following manufacturer's instructions and subsequently stained for 30 min at 4°C with fluorophore-coupled anti-human antibodies and/or fluorophore-coupled proteins. To stop the staining, cells were washed with PBS/1%BSA. DAPI was added before sorting to allow dead cell exclusion.

Co-culture of helper T cells with memory B cells:
Tonsillar follicular helper T cells (Tfh, CD19−CD4+CD45RA−CXCR5high) from patients who underwent routine tonsillectomy or peripheral memory T helper cells from bronchoalveolar lavage (BAL) of sarcoidosis patients (mostly peripheral helper T cells, Tph, CD19−CD4+CD45RA−) were sorted on an ARIA II flow cytometry sorter (Becton Dickinson). Patient samples were obtained from the Unfallkrankenhaus Marzahn (tonsils) or the Charité Universitätsmedizin Berlin (BAL). Sorted T cells were co-cultured for 7 days with heterologous tonsillar memory B cells (CD19+CD4−IgD−CD38−) at a 1:1 ratio in the presence of 4 ng/ml staphylococcal enterotoxin B (Toxin Technology) as described previously36. To block T-cell help, 20 µg/ml anti-CD40L

	antibody (clone TRAP1) and/or 10 µg/ml recombinant soluble IL-21 receptor (R&D Systems, Cat. 9249-R2) were added to the culture.
Instrument	Sortings were preformed using a MA900 Multi-Application Cell Sorter (Sony Biotechnology). Cell counting was performed using a MACSQuant16 flow cytometer (Miltenyi Biotec). Flow cytometry analysis was performed using a BD FACS Fortessa (BD Biosciences).
Software	Flow cytometry data was aquired using the software of a Multi-Application MA900 Cell Sorter (Sony Biotechnology) and FACSDiva (BD Biosciences), and analysed using FlowJo software 10.7.1 (TreeStar).
Cell population abundance	Cell population abundance was highly variable among subjects. Sorted population purity was analyzed during post-sorting cell counting using a MACSQuant flow cytometer (Miltenyi Biotec).
Gating strategy	Bone marrow plasma cell sorting: SSC-A x FSC-A, FSC-A x FSC-H, DAPI-/CD3-/CD10-/CD14- x FSC-A, CD38high x CD138+ or SSC-A x FSC-A, FSC-A x FSC-H, DAPI-/CD3-/CD14- x FSC-A, CD38 x CD27+, SAV-Biot-Spike-PE- x SAV-Biot-Spike-PE-Cy7-, TT-A488- x TT-A467-, CD38high x CD27high<br><br>Spike-specific B cell sorting: SSC-A x FSC-A, FSC-A x FSC-H, DAPI-/CD3-/CD14- x FSC-A, CD38 x CD27+, SAV-Biot-Spike-PE+ x SAV-Biot-Spike-PE+<br><br>TT-specific B cell sorting: SSC-A x FSC-A, FSC-A x FSC-H, DAPI-/CD3-/CD14- x FSC-A, CD38 x CD27+, SAV-Biot-Spike-PE- x SAV-Biot-Spike-PE-Cy7-, TT-A488+ x TT-A467+<br><br>Peripheral blood plasmablast sorting: SSC-A x FSC-A, FSC-A x FSC-H, DAPI-/CD14- x CD3-, CD38high x CD27high<br><br>Total bone marrow plasma cell analysis: SSC-A x FSC-A, FSC-A x FSC-H, LD-/CD3-/CD14- x CD19, CD38high x CD138+ (CD38 x CD19 and IgG x IgM)<br><br>RBD-specific bone marrow plasma cell analysis: SSC-A x FSC-A, FSC-A x FSC-H, LD-/CD3-/CD14- x CD19, CD38high x CD138+, RBD-A488 x RBD-A647 (CD38 x CD19 and IgG x IgM)<br><br>TT-specific bone marrow plasma cell analysis: SSC-A x FSC-A, FSC-A x FSC-H, LD-/CD3-/CD14- x CD19, CD38high x CD138+, TT-A488 x TT-A647 (CD38 x CD19 and IgG x IgM)<br><br>Tonsillar follicular helper T cells: DAPI- x FSC-A, FSC-H x FSC-A, SSC-H x SSC-A, CD4+ x CD19-, CD45RA– x CXCR5high<br><br>Tonsillar memory B cells: DAPI- x FSC-A, FSC-H x FSC-A, SSC-H x SSC-A, CD4– x CD19+, IgD– x CD38–<br><br>BAL peripheral memory T helper cells: DAPI- x FSC-A, FSC-H x FSC-A, SSC-H x SSC-A, CD4+ x CD19-, CD45RA–<br><br>Co-culture plasmablasts: DAPI- x FSC-A, FSC-H x FSC-A, SSC-H x SSC-A, CD19+ x CD4–, CD27+ x CD38+

☒ Tick this box to confirm that a figure exemplifying the gating strategy is provided in the Supplementary Information.
